# Environmental contamination of carbapenem-resistant gram-negative bacteria away from isolated patients in intensive care unit: a comprehensive surveillance study

**DOI:** 10.1038/s41598-025-30256-2

**Published:** 2025-12-30

**Authors:** Pan Xiao, Hui-Chao Huang, Chen Li, An-Hua Wu, Xun Huang, Chun-Hui Li, Fei-Yun Zhu, Si-Di Liu

**Affiliations:** 1 Medical Department, Liuyang Traditional Chinese Medicine Hospital, Changsha, China; 2 Infectious Diseases Department, Xiangya Hospital of Central South University, Changsha, China; 3 Clinical Laboratory Department, Liuyang Traditional Chinese Medicine Hospital, Changsha, China; 4 Science and Education Department, Liuyang Traditional Chinese Medicine Hospital, Changsha, China; 5 Infection Control Center, Xiangya Hospital of Central South University, 87 Xiangya Road, Changsha, 410008 China; 6 National Clinical Research Center for Geriatric Disorders, Xiangya Hospital of Central South University, Changsha, China

**Keywords:** Environmental contamination, Carbapenem-resistant gram-negative bacteria (CR-GNB), Intensive care unit (ICU), Nonisolated patients, Comprehensive surveillance, Infection control, Diseases, Health care, Medical research, Microbiology

## Abstract

Environmental contamination by carbapenem-resistant gram-negative bacteria (CR-GNB) contributes to healthcare-associated infections in intensive care unit (ICU). However, contamination in areas away from isolated patients remains inadequately characterized, and local epidemiological data are limited. A comprehensive surveillance study was conducted in the ICU of a tertiary hospital from January 2019 to December 2022. Monthly collections included clinical specimens (sputum, urine, blood) and environmental samples (inanimate surfaces, healthcare workers’ hands). Gram-negative bacteria were cultured, and carbapenem sensitivity testing was performed. Among 5,097 clinical specimens collected over the 4-year study period, 898 (17.6%) GNB isolates and 405 (8.0%) CR-GNB isolates were detected, with *Klebsiella pneumoniae* and carbapenem-resistant *Acinetobacter baumannii* (CRAB) being the most common species. From 2,215 environmental samples, 144 (6.5%) GNB and 90 (4.1%) CR-GNB strains were identified, predominantly *A. baumannii* and CRAB. The overall CR-GNB environmental contamination rates were highest on work coats (6.8%), followed by nonisolated patient medical devices (5.8%), nonisolated patient bed units (4.3%), mobile phones (4.1%), doctors’ offices and nurses’ stations (3.6%), and healthcare workers’ hands (2.0%). Spearman’s correlation analysis revealed a significant positive correlation between the monthly number of positive *A. baumannii* or CRAB isolates from environmental samples and clinical specimens. CR-GNB, particularly CRAB, contaminates ICU environments, including nonisolated patient areas and healthcare stations, highlighting the need for enhanced environmental surveillance in the ICU.

## Introduction

The gram-negative bacteria (GNB) *Klebsiella pneumoniae* (*K. pneumoniae*), *Acinetobacter baumannii* (*A. baumannii*) and *Pseudomonas aeruginosa* (*P. aeruginosa*) are among the “ESKAPE pathogens” that pose a great threat to antibiotic use and resistance in healthcare institutions. Carbapenems are key last-resort antimicrobial agents in the clinical field, as they are considered the treatment for multidrug-resistant (MDR) GNB infections^1^. With the increasing use of carbapenems, carbapenem-resistant gram-negative bacteria (CR-GNB) have become a major concern in healthcare-associated infections (HAIs)^2^. The prevalence of CR-GNB has increased since 2005, particularly among patients hospitalized in the intensive care unit (ICU)^3^. The high overall mortality rate (between 20% and 71%) among patients infected with CR-GNB poses a serious threat to clinicians^4^.

Nosocomial environmental contamination plays an important role in the transmission of HAIs^5,6^. It is estimated that 20% of HAIs originate from contaminated environmental surfaces^7^. The hospital environment acts as a significant reservoir for pathogens such as CR-GNB. Several pathogens (e.g., *A. baumannii*) can persist in the environment for extended periods, and molecular studies have confirmed homology between environmental and clinical isolates^8,9^. In recent years, transmissions caused by GNB, especially CR-GNB, have been observed in hospitals worldwide; these infections cause substantial public health problems and pose serious challenges to infection control^10,11^.

Currently, regular surveillance of nosocomial CR-GNB environmental contamination is inadequate, except for during outbreaks or suspected outbreaks of HAIs caused by CR-GNB for epidemiological investigations. In addition, most studies^12,13^ on nosocomial environmental contamination have focused on the environment of CR-GNB carriers, who are categorized as isolated patients and require contact precautions, but few studies have reported on environmental contamination away from isolated patients. Therefore, we conducted environmental and clinical surveillance of CR-GNB in the ICU over a 4-year period to assess the rate of environmental contamination of GNB and CR-GNB strains in the environment away from isolated patients as well as to determine the proportion of GNB and CR-GNB strains to provide information for infection prevention and control (IPC) and appropriate interventions in the ICU.

## Methods

### Study setting

This study was conducted from January 2019 to December 2022 in the intensive care unit of a 1200-bed tertiary teaching hospital in China. The ICU had 10 rooms with 21 beds until August 2022, when it was expanded to 15 rooms with 31 beds.

### IPC measures

To stop the spread of CR-GNB (CRKP, CRAB, or CRPA) in the hospital, patients with CR-GNB detected on admission or during hospitalization are categorized as isolated patients and require contact precautions. Contact precautions should include single-room isolation or patient cohorting. Conversely, nonisolated patients are those who tested negative for CR-GNB at admission and throughout their hospitalization.

Environmental services staff perform routine and terminal cleaning and disinfection in ICU. Bed units, medical devices, doctors’ offices and nurses’ stations should be cleaned and disinfected with 500 mg/L chlorine-containing disinfectant (Hunan Zhongda Zhongya Medical Technology Co., Ltd.) twice daily. The protocol of routine cleaning and disinfection begins with doctors’ offices and nurses’ stations, followed by bed units and medical devices for non-isolated patients, and finally bed units and medical devices for isolated patients. We provide pre-service training for environmental services staff in routine and terminal disinfection of environmental surfaces in the ICU, as well as 1–2 training per year. The Infection Control Center employs the Adenosine Triphosphate (ATP) Bioluminescence Monitoring System (Hygiena, USA) quarterly to verify environmental cleaning and disinfection efficacy. The established pass thresholds are: post-disinfection monitoring < 100 relative light units (RLU), and during-use monitoring < 300 RLU.

The ICU has implemented a rigorous prospective hand hygiene monitoring programme. Under this scheme, trained infection control nurses observe approximately 60 hand hygiene opportunities per month across the entire ICU, utilising the World Health Organisation’s “Five Key Moments for Hand Hygiene” framework. Hand hygiene compliance rates are calculated as the percentage of observed opportunities where the procedure was actually performed.

### Clinical specimens and isolates

Routine clinical specimens, including sputum, urine, and blood, were collected for diagnostic purposes from patients admitted to the ICU between 2019 and 2022. *K. pneumoniae*, *A. baumannii* and *P. aeruginosa* strains were isolated from clinical specimens using standard microbiology laboratory procedures and identified and tested for drug susceptibility on a fully automated Vitek 2 COMPACT microbiology system (BioMerieux, Craponne, France). Antimicrobial susceptibility testing was performed on Mueller-Hinton agar according to the Clinical and Laboratory Standards Institute (CLSI) M100 performance standards (2019–2022 editions). Duplicate strains were defined as the same bacterial species from the same specimen source from the same patient, and only the first strain of the duplicate strains was included in the analysis. The quality control strains used were the standard strains purchased from the Ministry of Health and included *K. pneumoniae* ATCC 700324, *A. baumannii* ATCC 17978, and *P. aeruginosa* ATCC 27853.

Carbapenem-resistant *K. pneumoniae* (CRKP), carbapenem-resistant *A. baumannii* (CRAB), and carbapenem-resistant *P. aeruginosa* (CRPA) were defined as isolates resistant to imipenem or meropenem based on the aforementioned CLSI guidelines.

### Environmental sampling

Environmental samples were randomly collected from inanimate surfaces and the hands of healthcare workers (HCWs) at the end of each month, with the exception of February 2020 due to the COVID-19 outbreak in China. Each month, 45 samples were taken (increased to 65 samples from August 2022 onward) from the ICU. The following environmental surfaces were frequently swabbed: (1) bed units for nonisolated patients (e.g., bed rail, bedside table, call button, patient record folder); (2) medical devices for nonisolated patients (e.g., infusion pump, touch pad for vital signs, touch pad or tubing surface for ventilators, stethoscope); (3) doctors’ offices and nurses’ stations (e.g., hand washing sink, computer keyboard or mouse, printer, landline telephone, drinking fountain, microwave); and (4) HCWs’ work coats, mobile phones, and hands.

The Hygiene Standard for Infections in Hospitals (GB 15982 − 2012) was followed when the sampling procedure was carried out for both inanimate surfaces and hand samples. A sterile cotton swab moistened with physiological saline (0.9% w/v) was moved back and forth over the surface of the inanimate object five times while the swab was rotated. For larger areas, the wiped area was 100 cm^2^; for areas below this threshold, the entire area was sampled. When collecting samples from the hands of HCWs before they washed them, a sterile cotton swab moistened with normal saline was passed twice over the flexed surfaces of the fingers of both hands from the base of the fingers to the fingertips while the swab was rotated. The portion touched by the collector’s hand was removed, and the remaining portion was placed in a test tube containing 10 ml of sterile saline.

### Environmental Microbiological methods

One milliliter of sample solution was taken from the well-shaken test tube to inoculate the brain heart infusion (BHI) agar plate, and the plate was incubated at 37 °C for 48 h. The isolates on the BHI agar plate were identified as *K. pneumoniae*, *A. baumannii*, or *P. aeruginosa* using a fully automated Vitek 2 COMPACT microbiology system (BioMerieux, Craponne, France). For antimicrobial susceptibility testing, the identified isolates were inoculated on Mueller-Hinton agar with two papers each containing 10 µg of imipenem or 10 µg of meropenem. CRKP, CRAB and CRPA were identified by the K-B paper disk diffusion test based on the CLSI M100 29th-32nd guidelines. Quality control was performed using the standard strains *K. pneumoniae* ATCC 700324, *A. baumannii* ATCC 17978, and *P. aeruginosa* ATCC 27853.

### Statistical analysis

The data were entered and analyzed using SPSS version 27. Categorical data are expressed as frequencies and percentages. Comparisons between groups were performed using the chi-square test, while the correlation between the number of positive GNB or CR-GNB isolated monthly from environmental and clinical samples was analyzed using Spearman’s correlations, with *p* < 0.05 considered to indicate a statistically significant difference.

## Results

### GNB and CR-GNB from clinical specimens

**Fig. 1:**
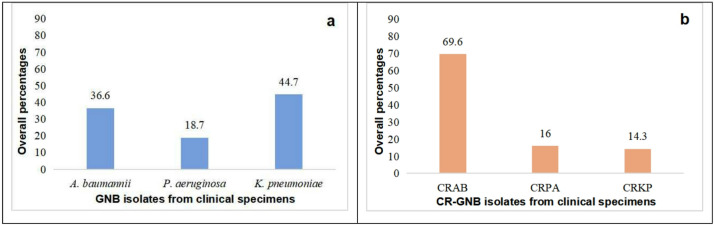
Overall percentages of GNB and CR-GNB isolates from the ICU clinical specimens from 2019 to 2022. GNB: gram-negative bacteria; CR-GNB: carbapenem-resistant gram-negative bacteria. (**a**): A comparison of *A. baumannii* (36.6%, 329/898), *P. aeruginosa* (18.7%, 168/898), and *K. pneumoniae* (44.7%, 401/898) isolated from clinical specimens revealed a statistically significant difference (χ^2^ = 142.640, *p* < 0.001). (**b**): A comparison of CRAB (69.6%, 282/405), CRPA (16.1%, 65/405), and CRKP (14.3%, 58/405) isolated from clinical specimens revealed a statistically significant difference (χ^2^ = 360.422, *p* < 0.001).

Between 2019 and 2022, 5,097 routine clinical specimens were collected for diagnostic purposes from patients admitted to the ICU. Among these, 898 (17.6%) GNB isolates were identified, predominantly comprising *K. pneumoniae* (401 strains). Additionally, 405 (8.0%) CR-GNB isolates were identified, with CRAB being the most prevalent (282 strains). Significant differences were observed in the distributions of both the three major GNB (χ2 = 142.640, *p* < 0.001; Fig. 1a) and the three major CR-GNB (χ2 = 360.422, *p* < 0.001; Fig. 1b) isolated from clinical specimens. Additionally, significant variations occurred in the isolation rates of A. *baumannii*, CRAB, and CRKP across the four study years (2019–2022), as presented in Table 1.

**Table 1:** GNB and CR-GNB isolated from clinical specimens in the ICU from 2019 to 2022, n (%).

Year	2019(*n* = 1193)	2020(*n* = 1161)	2021(*n* = 1210)	2022(*n* = 1533)	*p*
GNB	203 (17.0)	215(18.5)	187(15.5)	293(19.1)	0.066
*A. baumannii*	68(5.7)	76(6.5)	53(4.4)	132(8.6)	**< 0.001**
*P. aeruginosa*	31(2.6)	37(3.2)	48(4.0)	52(3.4)	0.306
*K. pneumoniae*	104(8.7)	102(8.8)	86(7.1)	109(7.1)	0.194
**CR-GNB**	92(7.7)	83(7.1)	75(6.2)	155(10.1)	**0.001**
CRAB	63(5.3)	64(5.5)	46(3.8)	109(7.1)	**0.002**
CRPA	10(0.8)	15(1.3)	23(1.9)	17(1.1)	0.116
CRKP	19(1.6)	4(0.3)	6(0.5)	29(1.9)	**< 0.001**

Note: GNB, gram-negative bacteria; CR-GNB, carbapenem-resistant gram-negative bacteria. Bold p-values indicate statistical significance (*p* < 0.05).

### GNB and CR-GNB from environmental samples

**Fig. 2:**
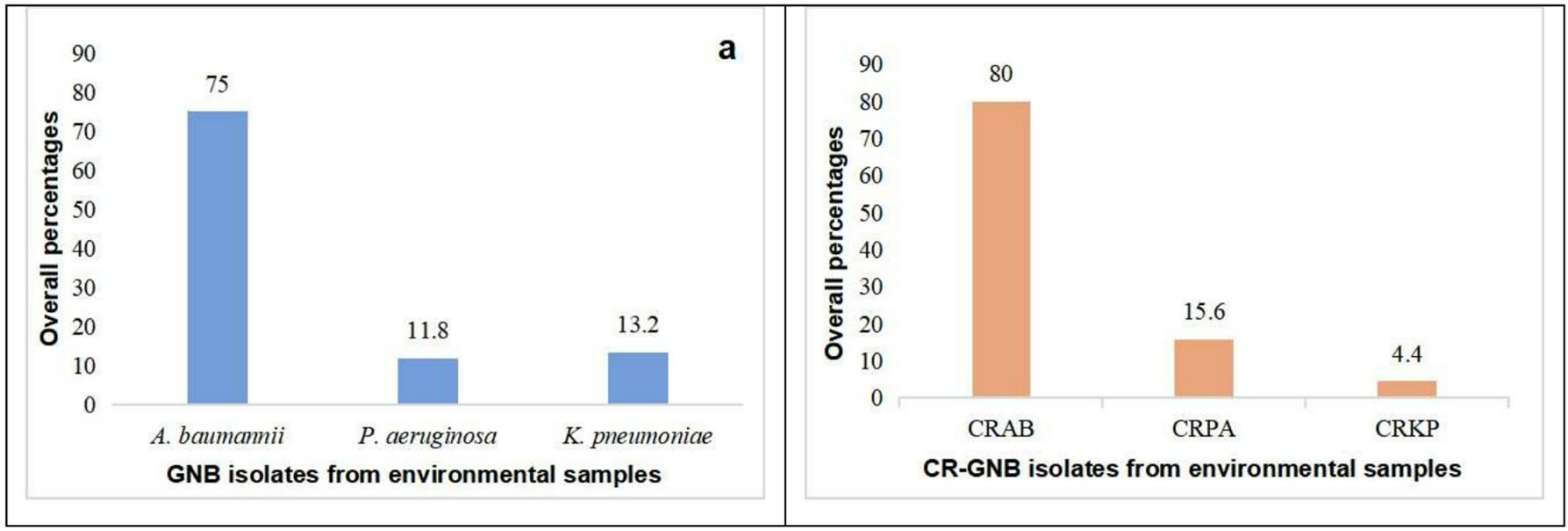
Overall percentages of GNB and CR-GNB isolates from the ICU environmental samples from 2019 to 2022. GNB: gram-negative bacteria; CR-GNB: carbapenem-resistant gram-negative bacteria. (**a**): A comparison of *A. baumannii* (75%, 108/144), *P. aeruginosa* (11.8%, 17/144) and *K. pneumoniae* (13.2%, 19/144) isolated from environmental samples revealed a statistically significant difference (χ^2^ = 168.813, *p* < 0.001). (**b**): A comparison of CRAB (80%, 72/90), CRPA (15.6%, 14/90), and CRKP (4.4%, 4/90) isolated from environmental samples revealed a statistically significant difference (χ^2^ = 134.800, *p* < 0.001).

From 2019 to 2022, 2,215 environmental samples were collected from inanimate surfaces and the hands of HCWs in the ICU. A total of 144 (6.5%) GNB were isolated, with *A. baumannii* predominating (75%; 108/144), followed by *K. pneumoniae* (13.2%; 19/144) and *P. aeruginosa* (11.8%; 17/144). A significant difference was observed in the percentages of the three GNB (χ2 = 168.813, *p* < 0.001; Fig. 2a). Similarly, 90 (4.1%) CR-GNB were detected, comprising CRAB (80%, 72/90), CRPA (15.6%, 14/90), and CRKP (4.4%, 4/90), with significant variation among these CR-GNB types (χ2 = 134.800, *p* < 0.001; Fig. 2b). Temporal analysis revealed significant yearly variations in most isolates’ prevalence (2019-2022), except for K. *pneumoniae* (χ2 = 3.977, *p* = 0.264) and CRKP (χ2 = 0.035, *p* = 0.998) (Table 2).

**Table 2:** GNB and CR-GNB strains isolated from the ICU environments from 2019 to 2022, n (%).

Year	2019(*n* = 540)	2020^*^ (*n* = 495)	2021(*n* = 540)	2022^#^(*n* = 640)	*p*
**GNB**	18 (3.3)	48 (9.7)	31 (5.7)	47 (7.5)	**< 0.001**
*A. baumannii*	13 (2.4)	32 (6.5)	28 (5.2)	35 (5.5)	**0.016**
P. *aeruginosa*	2 (0.4)	10 (2.0)	1 (0.2)	4 (0.6)	**0.003**
K. *pneumoniae*	3 (0.6)	6 (1.2)	2 (0.4)	8 (1.3)	0.264
**CR-GNB**	5 (0.9)	35 (7.1)	22 (4.1)	28 (4.4)	**< 0.001**
CRAB	3 (0.6)	25 (5.1)	21 (3.9)	23 (3.6)	**< 0.001**
CRPA	1 (0.2)	9 (1.8)	0	4 (0.6)	**0.001**
CRKP	1 (0.2)	1 (0.2)	1 (0.2)	1 (0.2)	0.998

Note: GNB, gram-negative bacteria; CR-GNB, carbapenem-resistant gram-negative bacteria. Bold p-values indicate statistical significance (*p* < 0.05).

*We did not conduct environmental sampling in the ICU in February 2020 due to the COVID-19 outbreak in China.

^#^The number of environmental samples in the ICU increased from 45 to 65 per month beginning in August 2022 due to bed capacity expansion.

### Detection rates of CR-GNB in clinical and environmental samples

The detection rates of carbapenem-resistant Gram-negative bacteria were 45.1% in clinical and 62.5% in environmental samples. Among these, CRAB showed persistently high resistance in both settings (85.7% vs. 66.7%). Notably, the environmental resistance proportions for CRPA and CRKP were 82.4% and 21.1%, respectively, which were elevated compared to their clinical rates of 38.7% and 14.5%.

**Table 3:** Detection rates of CR-GNB in clinical and environmental samples from 2019 to 2022, n (%).

CR-GNB detection rate	2019	2020	2021	2022	Total
Clinical					
CR-GNB	92/203 (45.3)	83/215 (38.6)	75/187 (40.1)	155/293 (52.9)	405/898 (45.1)
CRAB	63/68 (92.7)	64/76 (84.2)	46/53 (86.8)	109/132 (82.6)	282/329 (85.7)
CRPA	10/31 (32.3)	15/37 (40.5)	23/48 (47.9)	17/52 (32.7)	65/168 (38.7)
CRKP	19/104 (18.3)	4/102 (3.9)	6/86 (7.0)	29/109 (26.6)	58/401 (14.5)
Environmental					
CR-GNB	5/18 (27.8)	35/48 (72.9)	22/31 (71.0)	28/47 (59.6)	90/144 (62.5)
CRAB	3/13 (23.1)	25/32 (78.1)	21/28 (75.0)	23/35 (65.7)	72/108 (66.7)
CRPA	1/2 (50.0)	9/10 (90.0)	0/1 (0)	4/4 (100.0)	14/17 (82.4)
CRKP	1/3 (33.3)	1/6 (16.7)	1/2 (50.0)	1/8 (12.5)	4/19 (21.1)

Note: CR-GNB detection rate = (number of CR-GNB/total number of GNB isolates) ×100.0%.

### Sampling sites in GNB- and CR-GNB- positive environments

Environmental surveillance conducted in the ICU between 2019 and 2022 identified 144 GNB isolates (positive rate: 6.5%) and 90 CR-GNB isolates (positive rate: 4.1%). As detailed in Table 4, contamination distribution analysis revealed that bed units of nonisolated patients accounted for 8.3% of GNB isolates (4.3% CR-GNB), medical devices of nonisolated patients for 7.0% (5.8% CR-GNB), doctors’ offices or nurses’ stations for 5.9% (3.6% CR-GNB), HCWs’ work coats for 9.1% (6.8% CR-GNB), mobile phones for 6.1% (4.1% CR-GNB), and hands for 5.0% (2.0% CR-GNB).

The detection rates of GNB and CR-GNB in the bed units of nonisolated patients were lower in rooms after terminal disinfection (7.6% and 3.4%) than in rooms occupied by patients (8.7% or 4.7%), though differences were not statistically significant (GNB: χ2 = 0.128, *p* = 0.720; CR-GNB: χ2 = 0.367, *p* = 0.545). Similarly, no significant differences emerged between in-use and disinfected medical devices for GNB (8.3% vs. 4.6%; χ2 = 1.497, *p* = 0.221) or CR-GNB (7.3% vs. 2.8%; χ2 = 2.794, *p* = 0.095). Notably, hand washing sink in doctors’ offices and nurses’ stations exhibited the highest contamination rates (GNB:19.6%; CR-GNB:16.3%), significantly greater than that non-hand washing sinks (GNB: χ2 = 33.546, *p* < 0.001; CR-GNB: χ2 = 46.258, *p* < 0.001). *P. aeruginosa* (14 strains) and CRPA (12 strains) predominanted in hand washing sinks.

**Table 4:** GNB- and CR-GNB-positive environmental sampling sites in the ICU, 2019-2022, n (%).

Sampling sites	Number of samples	GNB				CR-GNB			
		A. baumannii	*P*. aeruginosa	K. pneumoniae	Total	CRAB	CRPA	CRKP	Total
**Bed units for nonisolated patients**	373	25 (6.7)	0	6 (1.6)	31 (8.3)	14 (3.8)	0	2 (0.5)	16 (4.3)
Patient-occupied rooms	254	18 (7.1)	0	4 (1.6)	22 (8.7)^*^	11 (4.3)	0	1 (0.4)	12 (4.7)^*^
Unoccupied room after terminal disinfection	119	7 (5.9)	0	2 (1.7)	9 (7.6)^*^	3 (2.5)	0	1 (0.8)	4 (3.4)^*^
**Medical devices for nonisolated patients**	327	21 (6.4)	0	2 (0.6)	23 (7.0)	19 (5.8)	0	0	19 (5.8)
In-use devices	218	16 (7.3)	0	2 (0.9)	18 (8.3)^†^	16 (7.3)	0	0	16 (7.3)^†#^
Disinfected devices	109	5 (4.6)	0	0	5 (4.6)^†^	3 (2.8)	0	0	3 (2.8)^†^
**Doctors’ offices and nurses’ stations**	1222	49 (4.0)	15 (1.2)	8 (0.7)	72 (5.9)	31 (2.5)	12(1.0)	1 (0.1)	44 (3.6)
Hand washing sinks	92	3 (3.3)	14 (15.2)	1 (1.1)	18 (19.6)^‡^	2 (2.2)	12(13.0)	1 (1.1)	15 (16.3)^‡**^
Non-hand washing sinks	1130	46 (4.1)	1 (0.1)	7 (0.6)	54 (4.8)^‡^	29 (2.6)	0	0	29 (2.6)^‡^
**Work coats**	44	3 (6.8)	1(2.3)	0	4 (9.1)	2 (4.5)	1(2.3)	0	3 (6.8)
**Mobile phones**	148	8 (5.4)	0	1 (0.7)	9 (6.1)	6 (4.1)	0	0	6 (4.1)
**Hands**	101	2 (2.0)	1 (1.0)	2 (2.0)	5 (5.0)	0	1(1.0)	1 (1.0)	2 (2.0)
**Total**	2215	108 (4.9)	17 (0.8)	19 (0.9)	144 (6.5)	72 (3.3)	14(0.6)	4 (0.2)	90 (4.1)

*Note: GNB*,* gram-negative bacteria; CR-GNB*,* carbapenem-resistant gram-negative bacteria. Percentages are calculated as (n/N)*100, where N is the number of samples for each site. Statistical comparisons (chi-square test): *Patient-occupied vs. terminal disinfection rooms: GNB (*p* = 0.720), CR-GNB (*p* = 0.545); †In-use vs. disinfected medical devices: GNB (*p* = 0.221), CR-GNB (*p* = 0.095); ‡Hand washing sinks vs. other surfaces: GNB (*p* < 0.001), CR-GNB (*p* < 0.001).

### Correlations between the number of positive GNB or CR-GNB strains isolated from environmental and clinical samples per month

**Fig. 3:**
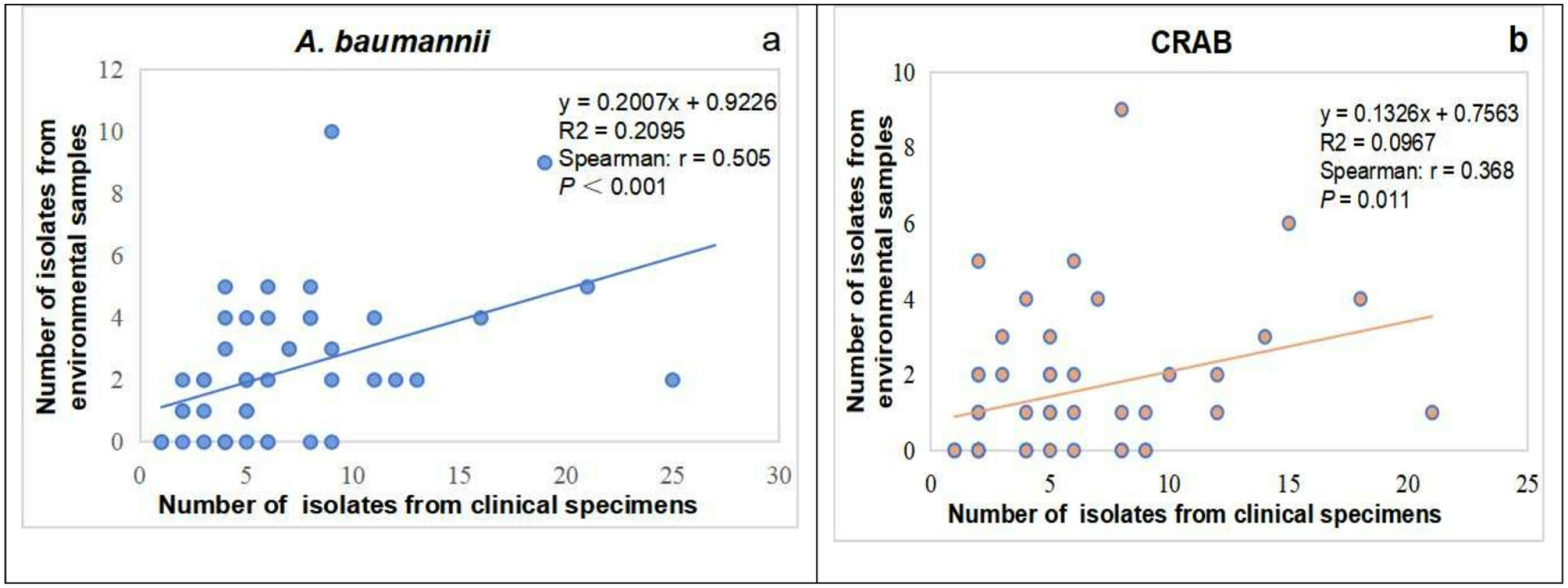
Correlations between the monthly numbers of positive GNB or CR-GNB isolated from environmental and clinical samples according to Spearman’s correlation analysis.

A significant positive correlation was observed between the number of positive *A. baumannii* (Spearman’s rho = 0.505, *p* < 0.001; Fig. 3a) isolated from environmental samples and clinical specimens per month. Notably, this association was similarly pronounced for CRAB (Spearman’s rho = 0.368, *p* = 0.011;Fig. 3b). However, this significant positive correlation was not observed in other GNB (*P. aeruginosa* and *K. pneumoniae*) or other CR-GNB (CRPA and CRKP).

## Discussion

Our study is the first 4-year comprehensive surveillance study to describe and analyze all isolated GNB and CR-GNB strains from environmental and clinical samples in the ICU of a tertiary university medical center in Changsha. Our results revealed that GNB and even CR-GNB can be detected in nonisolated patient environments and devices as well as in doctors’ offices and nurses’ stations, with CRAB being the predominant CR-GNB. In other words, GNB and CR-GNB contaminate the ICU through cross-transmission. The issue of environmental contamination may pose an even greater challenge in the ICU, where immunocompromised critically ill patients are often subjected to a variety of invasive diagnostic procedures and treatments^15^.

Few studies have examined environmental contamination outside of isolated patient areas. However, one study that sampled the surroundings of patients with positive *CRAB* clinical cultures within 7 days found a contamination rate ranging from 19% to 60.7%^15^, which is significantly higher than the contamination rate in environments outside isolated patient areas (CR-GNB: 4.1%) in our study. Additionally, Li K et al. conducted dynamic monitoring over the course of a year using 16 S rRNA sequencing to analyze bed units and medical devices before disinfection, revealing that *Acinetobacter* was consistently abundant in ICU environments^16^. When HCWs tend to isolated and nonisolated patients or walk from a contaminated hospital environment to the vicinity of nonisolated patients, they can potentially spread bacteria horizontally through their hands. The environment is a reservoir of microorganisms that can be transmitted to nonisolated patients via hands and invasive devices. Thus, to stop the spread of GNB, especially CR-GNB, in the ICU, environmental facility disinfection and stringent hand hygiene are crucial for HCWs.

However, after four years of environmental surveillance, we found that GNB could be isolated from disinfected bed units and medical devices of nonisolated patients. Furthermore, there was no statistically significant difference in the detection rate of either GNB or CR-GNB between the pre- and postdisinfection periods. This is believed to be due to insufficient disinfection, which is typically performed through manual wiping with disinfectants and wipes for bed units and medical devices in our ICU. On the one hand, a study has shown that it is difficult to remove or eradicate environmental bacteria through contact disinfection of the environment^17^. Currently, an increasing number of studies have shown that ultraviolet light disinfection is superior to environmental contact disinfection and reduces environmental contamination^18,19^. Consequently, the use of no-touch environmental disinfection, for example, with ultraviolet light, is recommended for the terminal disinfection of bed units in ICU. On the other hand, low pay, heavy workloads, and high staff turnover among environmental services staff have contributed to lapses in adhering to cleaning and disinfection protocols. Ensuring a stable workforce, providing adequate supplies and equipment, and offering more training and supervision can improve compliance and reduce HAIs^20^. These factors will be the focus of our subsequent IPC program.

Hand hygiene is a cost-effective, convenient and effective way to protect individuals and prevent the transmission of pathogens^21^. However, we isolated 5% (5/101) of the GNB strains from the hands of HCWs, with one CRAB strain and one CRKP strain. This rate is consistent with the isolation rate of GNB from the hands of HCWs (7.6%) reported in a previous study but with different causative organisms (mainly *P. aeruginosa*)^22^.Notably, despite surveillance data showing an overall hand hygiene compliance rate of 76.0% (with annual fluctuations ranging from 71.7 to 84.2%) during the study period, the detection rates of GNB and CR-GNB on the mobile phones of HCWs were 5.4% and 4.1%, respectively, predominantly *A. baumannii.* A study on bacterial colonization on mobile phones and hands of HCWs showed that four (3.6%) and five (4.5%) strains of *A. baumannii* were detected on these, respectively, indicating cross-contamination between mobile phones and hands of HCWs^23^. In addition, we isolated 72 strains of GNB, 44 of which were CR-GNB, from doctors’ offices and nurses’ stations; the surfaces included hand washing sinks, computer keyboards or mice, printers, landline telephones, drinking fountains, and microwaves. HCWs may be less likely to adhere to hand hygiene guidelines when working in nonpatient areas such as doctors’ offices or nurses’ stations, which are perceived to be safer than patient environments. Therefore, strict hand hygiene is vital, and the disinfection of mobile phones must be taken seriously in the ICU.

A 2021 report from the China Antimicrobial Resistance Surveillance System (CARSS) stated that *K. pneumoniae*, *A. baumannii*, *Escherichia coli*, and *P. aeruginosa* were the most common clinical isolates from the ICU. The resistance rates of these four bacteria to carbapenems were 20.6%, 78.2%, 3.5%, and 28.7%, respectively^24^. This finding is comparable to that in our clinical study, which showed that the GNB isolates and the CR-GNB isolates from clinical specimens were dominated by *K. pneumoniae* and CRAB, respectively. However, the predominant GNB isolated from environmental samples did not coincide with the clinical specimens in this study, and the most common isolate from the environmental samples was *A. baumannii*. This conclusion conflicts with those of previous studies in which *K. pneumoniae* or *P. aeruginosa* were the main GNB isolated from the surrounding environment and equipment^25,26^; however, these findings are consistent with those of other studies on contaminating bacteria in the ICU^27–29^. The isolation of distinct bacteria from surfaces in various healthcare settings may be connected to locally prevalent strains as well as the type of objects or equipment sampled. As in this study, hand washing sinks were the primary site of *P. aeruginosa* detection, while other objects or devices were the primary site of *A. baumannii* detection. Given that most of the limited *P. aeruginosa* isolates were obtained from washbasins, this sampling bias may explain the notably high detection rate of CRPA.

Finally, we further found a positive correlation between the number of *A. baumannii* or CRAB strains isolated per month from both the environmental and clinical samples via Spearman’s correlation analysis. In other words, the greater the number of patients with positive *A. baumannii* or CRAB isolated, the more contaminated the environment was with *A. baumannii* or CRAB. Notably, despite maintaining 85–90% compliance with surface cleaning protocols in the hospital during the study period, persistent CRAB contamination was still observed. The predominance of *A. baumannii* in the hospital environment is likely attributable to its remarkable capacity to survive on dry surfaces and its typically resistance to common disinfectants^30,31^. Based on these findings, we recommend implementing enhanced disinfection measures in areas surrounding wards treating CRAB-positive patients.

We also note that the observed increase in clinical CR-GNB isolates from 2020 coincides with multiple reports documenting a rise in healthcare-associated infections and antimicrobial resistance during the COVID-19 pandemic^32–34^. This trend is logically extended to our environmental findings: the concurrent rise in environmental CR-GNB isolates likely reflects this heightened clinical prevalence, as an expanded reservoir of colonized or infected patients would be expected to contribute to increased environmental contamination.

This study had several limitations. First, the absence of molecular genotyping for environmental and clinical isolates precludes confirmation of their clonal relatedness, despite the observed significant temporal correlations. Furthermore, the classification of “non-isolated” areas might have been influenced by undetected patient colonization. Second, *E. coli* was not included among GNB in the present study due to the low detection rate of carbapenem-resistant *E. coli* in clinical specimens. Third, as a single-center study conducted in a regional core hospital, the generalizability of our findings may be limited since the distribution and prevalence patterns of CR-GNB exhibit significant geographic variation across different regions and healthcare settings. Finally, the absence of recognized outbreaks linked to the target pathogens meant that no targeted interventions (e.g., no-touch disinfection or sink upgrades) were introduced. This lack of escalation in control measures may have contributed to the environmental persistence and the observed pattern of endemic circulation of CR-GNB in the ICU; however, we plan to implement improvements to reduce environmental contamination and cross-infection based on the key findings of this study.

## Conclusions

In summary, this study primarily demonstrated that CR-GNB, particularly CRAB, contaminate the ICU environment beyond the immediate vicinity of isolated patients, likely mediated through cross-transmission. The prevalence and clinical significance of these environmentally persistent strains within hospitals remain inadequately characterized. Given the immunocompromised status of ICU patients and their frequent requirement for invasive procedures, we recommend implementing environmental contamination monitoring for CR-GNB (with emphasis on CRAB) in ICU settings. Key monitoring targets should include bed units, medical devices, and sinks to guide and evaluate the effectiveness of targeted cleaning and disinfection protocols.

## Data Availability

Data is provided within the manuscript.

## Abbreviations

*A. baumannii*: *Acinetobacter baumannii*
ATP: Adenosine Triphosphate
BHI: Brain heart infusion
CLSI: Clinical Laboratory Standards Institute
CRAB: Carbapenem-resistant *Acinetobacter baumannii*
CR-GNB: Carbapenem-resistant gram-negative bacteria
CRKP: Carbapenem-resistant *Klebsiella pneumoniae*
CRPA: Carbapenem-resistant *Pseudomonas aeruginosa*
GNB: Gram-negative bacteria
HAIs: Healthcare-associated infections
HCWs: Healthcare workers
ICU: Intensive care unit
IPC: Infection prevention and control
*K. pneumonia*: *Klebsiella pneumoniae*
*P. aeruginosa*: *Pseudomonas aeruginosa*
RLU: Relative light units
